# Electrochemical oxidation of surfactants as an essential step to enable greywater reuse

**DOI:** 10.1016/j.eti.2024.103563

**Published:** 2024-05

**Authors:** Alexsandro J. dos Santos, Hongchen Shen, Marcos R.V. Lanza, Qilin Li, Sergi Garcia-Segura

**Affiliations:** a Nanosystems Engineering Research Center for Nanotechnology-Enabled Water Treatment, School of Sustainable Engineering and the Built Environment, Arizona State University, Tempe, AZ 85287–3005, United States; b São Carlos Institute of Chemistry, University of São Paulo, Avenida Trabalhador São Carlense 400, São Carlos, SP 13566–590, Brazil; c Nanosystems Engineering Research Center for Nanotechnology-Enabled Water Treatment, Department of Chemical and Biomolecular Engineering, Department of Materials Science and NanoEngineering, and Department of Civil and Environmental Engineering, Rice University, MS 319, 6100 Main Street, Houston 77005, USA

**Keywords:** Electrochemical advanced oxidation processes, Sodium dodecyl sulfate, Water treatment, Water reuse, Greywater

## Abstract

The practical application of electrochemical oxidation technology for the removal of surfactants from greywater was evaluated using sodium dodecyl sulfate (SDS) as a model surfactant. Careful selection of electrocatalysts and optimization of operational parameters demonstrated effective SDS removal in treating a complex greywater matrix with energy consumption below 1 kWh g^−1^ COD (Chemical Oxygen Demand), paving the way for a more sustainable approach to achieving surfactant removal in greywater treatment when aiming for decentralized water reuse. Chromatographic techniques identified carboxylic acids as key byproducts prior to complete mineralization. These innovative approaches represent a novel pathway for harnessing electrochemical technologies within decentralized compact devices, offering a promising avenue for further advancements in this field.

## Highlights

• Complete mineralization of SDS can be achieved by Electrochemical oxidation (ECO).
• Energy efficiency decreases with increasing current density and decreasing SDS concentration.
• SDS mineralization is attributed to free radicals and reactive chlorine species.
• Carboxylic acids, previously not reported during SDS mineralization, are detected.

## Graphical Abstract

**Figure:**
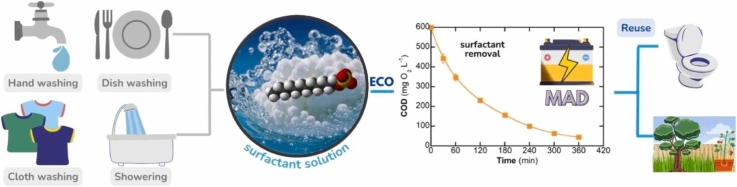

## 1 Introduction

The sustainable development goals (SDG) defined by the United Nations (UN) identify access to clean water and sanitation as one of the major challenges that humankind needs to overcome. However, this goal is far from being reached by 2030 (Fritz et al., 2019, Bleischwitz et al., 2018). The UN’s last reports estimate that nearly 130 countries may not attain this SDG of providing sustainably managed water resources for all. Progress is undermined by multiple extra stressors such as the lack of centralized water infrastructure, disconnection from the water grid, and water shortage, which continues to test the endurance of communities in need. Modular adaptative decentralized (MAD) technologies might be a light of hope on the horizon for communities without established water infrastructure (Stoler et al., 2022, Wutich et al., 2023). The question standing is what solution is needed to effectively address this challenge? In this context, electrified treatment for MAD presents itself as a viable solution. Electrochemical water technologies, characterized by being both low-cost and safe, offer the advantage of eliminating the need for chemical transportation and storage, thereby reducing associated risks.

Greywater comprises wastewater originating from all household sources except toilets. This includes water from appliances such as washing machines, dishwashers, showers, baths, and sinks (Friedler and Hadari, 2006). Developing countries exhibit an average greywater production higher than 200 liters per capita (Garrido-Baserba et al., 2022, Van de Walle et al., 2023). Implementing water reuse technologies could significantly reduce the water footprint per household by 40–80%, particularly if greywater is reused within the household to flush toilets (Noutsopoulos et al., 2018). This relevant water saving would alleviate water stressed communities and enable suitable practices that warrant access to clean water and sanitation for all. Greywaters are diverse in source and composition, but one common characteristic is their high organic load associated with the presence of surfactants. Sodium dodecyl sulfate (SDS), propylene glycol, trimethyl amine, and linear alkylbenzene sulphonate are commonly reported surfactants in greywaters (Al-Jayyousi, 2003, Shaikh and Ahammed, 2020, Shreya et al., 2021). Nevertheless, SDS is the most extensively used and representative species in greywaters; it generally represents ∼80% of the chemical oxygen demand (COD). Although toxicological data regarding SDS is relatively limited in the current literature, research findings have indicated that SDS can trigger lipid peroxidation, elevate glutathione production, and induce alterations in carbon metabolism (Singer and Tjeerdema, 1993, Sirisattha et al., 2004). Therefore, ensuring the removal of SDS is one of the key steps when considering greywater reuse and mitigating potential human health concerns.

As electrified water treatment technologies emerge as feasible solutions for environmental applications, some aspects should be pondered in the engineering decision-making process. When considering the idiosyncrasy and techno-economic peculiarities of different countries, one may find additional barriers to be surpassed(dos Santos et al., 2023). The disconnection from the electrical grid as well as the transition towards energy renewable sources must be balanced and contemplated. Ideal energy key performance indicators should set desirable energy requirements to increase the accessibility and feasibility of technology implementation in developing regions. Electrified water processes will become game changers and catalyze access to clean water for all if treatment can be attained using the daily electrical outputs of a single solar panel, which are estimated to be ca. 1.0 kWh day^−1^.

Electrochemical oxidation (ECO) is perhaps one of the most promising approaches for the decentralized treatment of organic contaminants (Chaplin, 2014, Ganiyu et al., 2016, Stirling et al., 2020). As an advanced oxidation process, ECO relies on the electrogeneration of highly reactive oxidizing agents, including hydroxyl radicals, singlet oxygen, and sulfate radicals (Brillas, 2022, Hu et al., 2021, Köktaş and Gökkuş, 2022, Xie et al., 2023). These species effectively target and degrade organic compounds, ultimately leading to their complete mineralization (Lu et al., 2022, Moreira et al., 2017, Ren et al., 2023). ECO has demonstrated capabilities to completely degrade recalcitrant compounds such as pharmaceuticals (Deng et al., 2022, Du et al., 2021, Loos et al., 2018, Yang et al., 2022, Zhang et al., 2021), endocrine disruptors (Cui et al., 2009, de Luna and Bensalah, 2022, Dietrich et al., 2017), pesticides (da Costa et al., 2021, Domínguez et al., 2021, Komtchou et al., 2017, Kronka et al., 2023, Samet et al., 2010) and dyes (Kaur et al., 2018, Nidheesh et al., 2018, Phetrak et al., 2020, Thiam et al., 2016, Zhang et al., 2014). However, studies assessing the applicability of ECO for greywater treatment are scarce. Therefore, this work aims to assess the feasibility of ECO technologies to enable greywater reuse based on the effective abatement of surfactant SDS. Engineering figures of merit are used to elucidate the situations where the deployment of decentralized ECO might be competitive as well as identifying the need to advance modular electrified treatments.

## 2 Materials and methods

### 2.1 Chemicals

Reagents of analytical grade were used to prepare all solutions. A synthetic surfactant solution contained sodium dodecyl sulfate (SDS, CAS number 151–21-3) and sodium sulfate (Na_2_SO_4_, CAS number 7757–82-6) which acted as a supporting electrolyte. A simulated greywater was prepared with sodium chloride (NaCl, CAS number 7647–14-5), calcium chloride (CaCl_2_, CAS number 10043–52-4), sodium nitrate (NaNO_3_, CAS number 7631–99-4), sodium phosphate monobasic (NaH_2_PO_4_, CAS number 7558–80-7) and ammonium bicarbonate (NH_4_HCO_3_, CAS number 213–911-5); all reagents were purchased from Sigma-Aldrich. Table 1 summarizes the amount by mass added of each reagent to prepare the greywater according to the model greywater composition that has been defined in the literature (Ghaitidak and Yadav, 2013). Sulfuric acid (H_2_SO_4_, CAS number 7664–93-9, Sigma-Aldrich) was used as a mobile phase for the high-performance liquid chromatography (HPLC) analysis to identify the by-products like short-carboxylic acids. All solutions were prepared with ultrapure water from an Elga Lab Water system.

**Table 1:** Inorganic greywater composition.

Chemical components	Values (mg L^−1^)
NaCl	181.3
CaCl_2_	55.5
NaNO_3_	137.0
NaH_2_PO_4_	201.0
NH_4_HCO_3_	163.7

### 2.2 Electrochemical set-up

Electrochemical assays were performed at 25 ºC in a lab-scale glass cell containing 100 mL of solution under continuous magnetic stirring at 600 rpm to ensure transport from/towards the electrodic surfaces. The electrolytic batch cell reactor was equipped with one anode and one cathode with an effective geometric area of 3.0 cm^2^. Electrodes were placed facing each other in parallel with an interelectrode distance of 1.5 cm. Stainless steel was used as the cathode in all experiments. Meanwhile, different electrocatalytic materials were evaluated as the anode, such as boron-doped diamond (BDD) thin film deposited on a Si substrate supplied by NeoCoat, pristine platinum (Pt 99.99%) provided by Stanford Advanced Materials, and a dimensional stable anode (DSA®) of IrO_2_ from De Nora. All experiments were performed at a fixed current density (*j*) provided by a TENMA 72–2720 DC power supply. Experiments were carried out in triplicate. Experimental results presented standard deviation below 5%.

### 2.3 Analytical methods

Chemical oxygen demand (COD) was measured at different reaction times by using the digester Hach model DRB200 for 2 h and then assessed by Hach UV–vis spectrophotometer DR 6000. The instantaneous current efficiency (ICE) and energy consumption per COD mass unit (kWh g⁻¹) were then computed using (1), (2) as follows (do Vale-Júnior et al., 2019).

(1) $$\%ICE=FVs\frac{\left({COD}_{t}-{COD}_{t+\Delta t}\right)}{8I\Delta t}x\,\;100$$

(2) $$\mathrm{Energy consumption}\left(\mathrm{kWh}{\left(\mathrm{g COD}\right)}^{-1}\right)=\frac{E_{\mathrm{cell}}I\;t}{1000V_{S}{(\mathrm{COD}}_{0}-\mathrm{COD})}$$

In the previous equations, the variables are defined as follows: *F* represents the Faraday constant (96,485 C mol⁻¹), *V*_*s*_ is the volume of the solution in liters, 8 stands for the oxygen equivalent mass (g eq⁻¹), *I* represents the applied current in amperes, *t* signifies the electrolysis time in seconds for ICE and hours for energy consumption and *E*_*cell*_ indicates the average potential difference between the electrodes in volts.

Electrochemical characterization of electrodes was conducted by cyclic voltammetry (CV). The electroanalyses by CV were carried out in a three-electrode system where the Pt plate was a counter electrode, Ag/AgCl (∼3 mol L^−1^ KCl) was used as the reference electrode, and the working electrode was either BDD, Pt, or IrO_2_. The CV analyses were performed in N_2_-purged 50 mM Na_2_SO_4_ solutions using an Autolab potentiostat/galvanostat (model PGSTAT128N) with a potential window ranging from 0 to 3.0 V vs. Ag/AgCl at a scan rate of 50 mV s^−1^.

Reaction products were analyzed using a Waters HPLC system (model e2695) equipped with a Bio-Rad Ion Exclusion Column Aminex HPX-87 H (300 mm 7.8 mm). Samples were run at 30 ºC with a 20 μL injection volume. Quantitative measurements were performed at 210 nm using a 2998 PDA detector. The mobile phase was 4.0 mM H_2_SO_4_ at a flow rate of 0.8 mL min^−1^. Carboxylic acids exhibited characteristic retention times (*t*_*r*_) of 6.1 min for oxalic acid, 8.5 min for tartaric acid, 9.8 min for malonic acid, 11.2 for succinic acid and 14.3 min for acetic acid. Inorganic species (NO_2_¯, NO_3_¯, and NH_4_^+^), total chlorine, and free chlorine were detected using Hach analytic kits.

## 3 Results and discussion

### 3.1 Electrocatalyst selection for improved surfactant removal

**Fig. 1:**
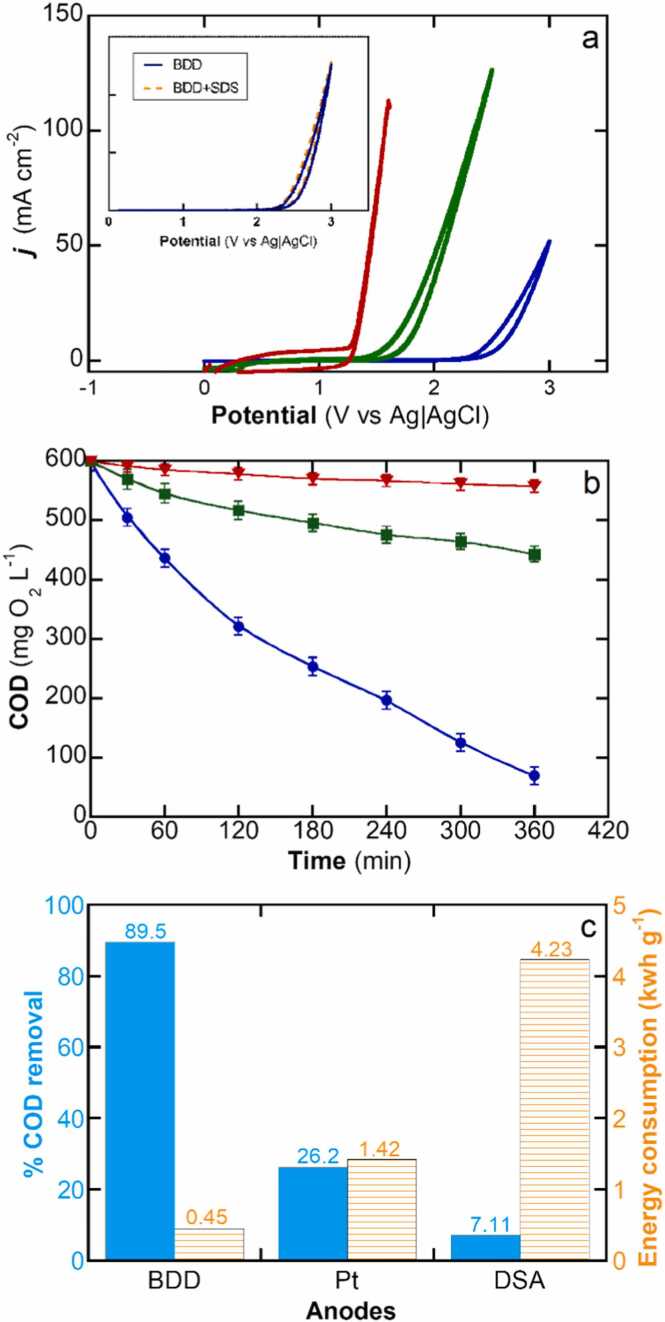
(a) Electrochemical response of different anode materials evaluated by cyclic voltammetry (CV) recorded at scan rate of 50 mV s^−1^ at pH 7.0 and 25 ºC. The inset panel in (a) shows the CV for BDD in () 50 mM Na_2_SO_4_ and in (⚫) 50 mM Na_2_SO_4_ + 10 mM SDS. (b) Electrochemical oxidation performance in terms of COD decay. (c) Shows the () percentage COD removal and () electrical energy consumption during the treatment of was 100 mL 300 mg L^−1^ SDS (600 mg L^−1^ COD) solution in 50 mM Na_2_SO_4_ (used as the supporting electrolyte) at pH 7.0 and 25 ºC. The applied current density *j* = 100 mA cm^−2^. Anodes: () IrO_2_, () Pt and () BDD.

Electrocatalytic processes are heterogeneous in nature and therefore driven by the unique properties of the electrode surface. The ECO process aims to use anodic materials that present high overpotential for the oxygen evolution reaction (OER) to stabilize metastable species such as reactive oxygen species (ROS) on their surfaces (da Costa et al., 2021, Zuo et al., 2023). The electrogenerated ROS from water oxidation (e.g., hydroxyl radical, superoxide radical, surface superoxide) are then responsible for recalcitrant organics oxidation and mineralization (Candia-Onfray et al., 2018, Clematis and Panizza, 2021, Olvera-Vargas et al., 2021). Fig. 1a illustrates the responses of the three electrocatalysts towards OER in cyclic voltammetry characterization. The IrO_2_ DSA® electrode presents an onset potential of 1.25 V, below that of the Pt (1.60 V) and BDD (2.50 V) electrodes. Furthermore, CV tests conducted in the presence of SDS, as depicted in the inset of Fig. 1a, reveal the absence of direct electron transfer mechanisms for SDS oxidation, as evident in the lack of any oxidation peaks prior to the onset of OER. These results support the predominance of a free radical reaction pathway in the ECO system.

Fig. 1b shows the abatement of COD during ECO treatment of a 300 mg L^−1^ SDS solution (600 mg L^−1^ COD), which is representative of the commonly found concentration of this surfactant in greywater (Ghaitidak and Yadav, 2013). Removal in presence of electrolyte but in absence of an applied current did not result in any COD abatement nor SDS removal. It can be seen that the BDD electrode, which has a higher OER overpotential, results in faster COD abatement. This behavior can be explained by the nature of the BDD electrode surface, which stabilizes physisorbed hydroxyl radical (see Eq.3), whereas Pt and IrO_2_ stabilizes this highly reactive species by chemisorption to form superoxides (Araújo et al., 2014, Jiang et al., 2021, Panizza and Cerisola, 2009, Pointer Malpass and de Jesus Motheo, 2021). Physisorbed hydroxyl radicals are more labile and reactive, which propitiates mineralization reactions as characteristic behaviors of so-called non-active electrodes (Montenegro-Ayo et al., 2023, Moreira et al., 2017). The electrogeneration of hydroxyl radicals on the BDD surface has been extensively addressed in the literature (Cai et al., 2019, Kapałka et al., 2007, Song et al., 2018). Conversely, the chemisorbed species induce chemical conversion but not mineralization, which is commonly observed in active electrodes. Furthermore, in a sulfate medium, the formation of sulfate radicals can occur to a limited extent through the direct oxidation of sulfate ions at the BDD electrode as shown in Eq. 4 (Cai et al., 2019, Song et al., 2018, Vernasqui et al., 2022).

(3) BDD + H_2_O → BDD(^•^OH) + H^+^ + e^−^

(4) SO_4_^2−^ → SO_4_^•−^+ e^−^

For these reasons, BDD electrodes may favor the abatement of the large COD loads of greywaters while effectively inducing the mineralization of surfactant SDS. The higher performance and suitability of BDD electrodes are summarized in Fig. 1c, where electrode performances are compared in terms of engineering figures of merit. Notably, BDD electrodes achieve a remarkable 13-fold higher COD abatement compared to IrO_2_ electrodes, while consuming only 0.45 kWh g^−1^ COD, which is four times lower than the Pt electrodes and fourteen times lower than the IrO_2_ DSA® electrode. Hence, we chose BDD electrodes as the electrocatalytic material for evaluating ECO performance.

### 3.2 Evaluating the impact of operational parameters

**Fig. 2:**
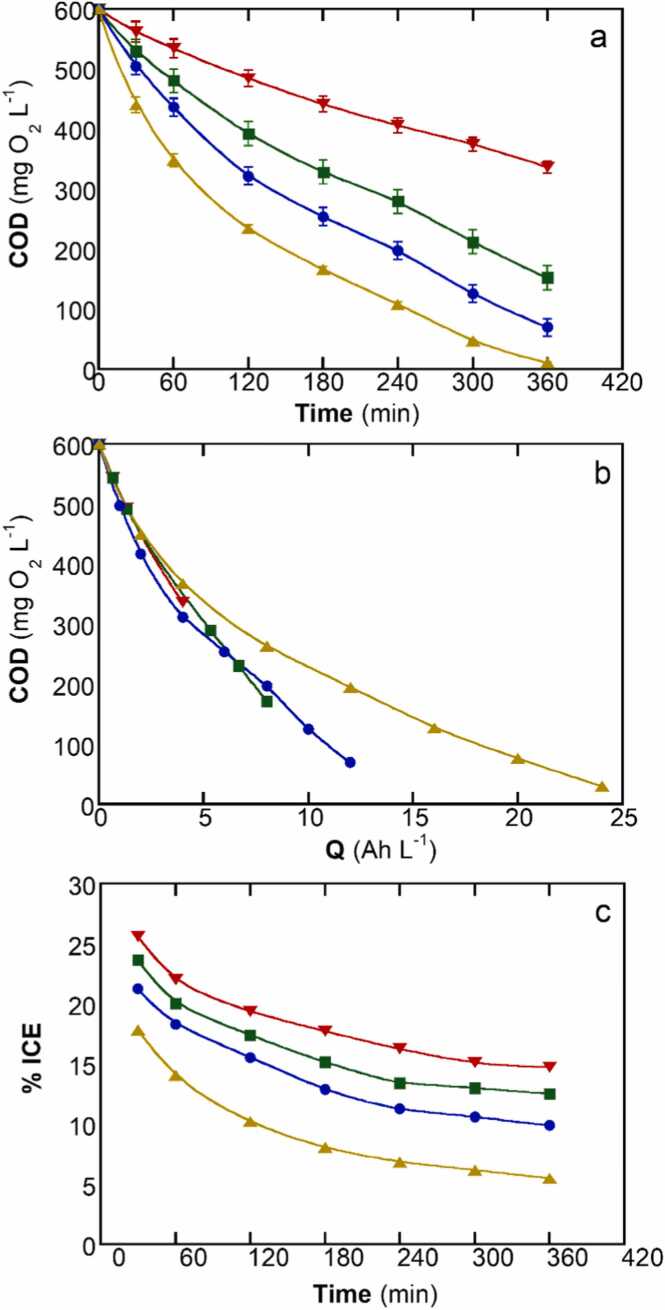
Influence of current density on COD decay (a) over time (b) over Q applied and (c) instantaneous current efficiency for the ECO process of 100 mL of 300 mg L^−1^ SDS (600 mg L^−1^ COD) solution in 50 mM Na_2_SO_4_ (used as the supporting electrolyte) at pH 7.0 and 25 ºC using a BDD anode. Current density: () 33.3 mA cm^−2^, () 66.7 mA cm^−2^, () 100 mA cm^−2^ and () 200 mA cm^−2^.

Experiments were conducted to optimize the performance of the operational parameters of ECO treatment. Among these parameters, current density *(j*) holds particular significance in electrified processes, as it influences the electrogeneration of free radicals and, consequently, the overall cost of the ECO. Fig. 2a clearly demonstrates a positive correlation between *j* values and the removal of SDS and its by-products. Over a 6-hour electrolysis process, the percentage of COD removal increased with increasing current density: 56.3% at 33.3 mA cm^−2^, 73.5% at 66.6 mA cm^−2^, 89.3% at 100 mA cm^−2^, and 96.8% at 200 mA cm^−2^. These results suggest that the higher *j* was more effective in abating surfactants. However, it is crucial to note that selecting an optimal *j* based solely on this information may not suffice as it does not consider energy efficiency. To better assess the process efficiency, COD removal versus applied electric charge (*Q*) is shown in Fig. 2b. The SDS mineralization increases with increasing delivered electric charge. The utilization efficiency of the electric charge remains nearly constant up to current densities of 100 mA cm^−2^, but was notably lower at 200 mA cm^−2^. Another approach to reaching the same conclusion involves analyzing the ICE, as depicted in Fig. 2c, that shows that ICE decreases with time; and ICE is lower at higher current densities. This phenomenon is linked to the enhancement of competitive reactions that consume electrogenerated oxidants (e.g., hydroxyl radicals). These competitive parasitic reactions include O_2_ evolution (Eq. 5) and, to a lesser degree, the generation of hydrogen peroxide through the dimerization of hydroxyl radicals (Eq. 6). It is important to note that hydrogen peroxide is generally regarded as a relatively weak oxidant, and it has been reported by several authors to be inefficient in the complete mineralization of organic pollutants (Kronka et al., 2023). Considering the efficient use of energy to drive the electrified water treatment, the optimal current density for effective SDS mineralization can be defined at 100 mA cm^−2^ as the condition that attains faster mineralization with lower energy requirements and higher ICE.

(5) BDD(^•^OH) → BDD + ½ O_2_ + H^+^ + e^−^

(6) 2 BDD(^•^OH) → 2 BDD + H_2_O_2_

**Fig. 3:**
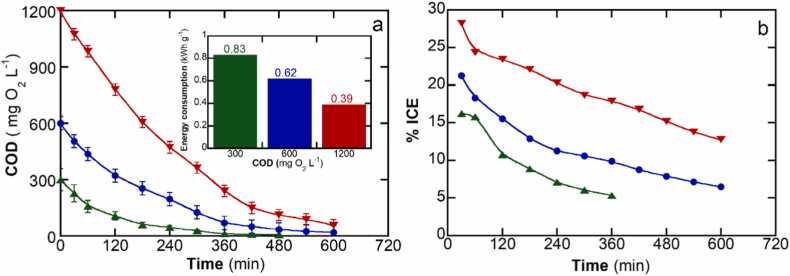
Effect of initial SDS concentration on (a) COD decay b) instantaneous current efficiency over time for the ECO process of 100 mL SDS solution in 50 mM Na_2_SO_4_ (used as the supporting electrolyte) at pH 7.0 and 25 ºC using a BDD anode applying *j* of 100 mA cm^−2^. SDS concentration: () 150 mg L^−1^ (300 mg L^−1^ COD), () 300 mg L^−1^ (600 mg L^−1^ COD) and () 600 mg L^−1^ (1200 mg L^−1^ COD). Inset represents the energy cost per COD unit.

In our study, another important parameter is the initial SDS concentration. This parameter holds particular significance due to the substantial variability in SDS concentrations across different greywater collection scenarios. Consequently, it is essential to know the concentration range within which ECO technology can demonstrate optimal efficiency. Electrolysis experiments were conducted using SDS concentrations spanning from 150 mg L^−1^ (300 mg COD L^−1^) to 600 mg L^−1^ (1200 COD mg L^−1^) with a treatment goal of at least 95% mineralization. As illustrated in Fig. 3a, a proportionally longer reaction time is necessary as the SDS concentration increases. On the other hand, energy consumption per unit mass of surfactant removal decreases with increasing initial SDS concentration, from 0.83 kWh g^−1^ COD at 150 mg L^−1^ SDS (300 mg L^−1^ COD) to 0.39 kWh g^−1^ COD at 600 mg L^−1^ SDS (1200 mg L^−1^ COD). It is important to note that the energy consumption remains well below 1 kWh g^−1^ COD across the wide range of SDS concentrations tested. In addition, Fig. 3b shows that higher SDS concentrations lead to higher energy efficiency. For instance, at the concentration of 600 mgL^−1^ of SDS, the ICE commenced at approximately 30% and concluded at 15% when reaching an SDS removal of 95%, whereas at the lower concentration of 150 mgL^−1^ of SDS, the ICE began at 16% and concluded at 5%. In accordance with existing literature, a plausible explanation for the ICE trend lies in the improvement of mass transport of SDS and its by-products from the bulk solution to the surface of the BDD electrode (Moreira et al., 2017). In other words, higher concentrations facilitate an enhancement in the ECO process. Conversely, as the concentration decreases, the extent of competitive parasitic reactions increases as the probability of oxidants to react with SDS in solution decreases. The concentration effects may arise as an important limitation for the electrochemical degradation of trace contaminants, such as those present at parts per billion (ppb) concentration levels (Garcia-Segura et al., 2020). Comparisons with other advanced oxidative processes reveal competitive SDS removal rates for ECO. For example, peroxy-electrocoagulation achieved 81.6% removal using iron as a sacrificial electrode with an initial SDS concentration of 60 mg L^−1^ (Yüksel et al., 2009). Photo-Fenton reached 95.4% for synthetic solutions and 71% for soft drink wastewater with an initial SDS concentration of 40 mg L^−1^ and UV-C intensity of 45 W (Malakootian et al., 2016). In the O_3_/UV/H_2_O_2_ process, an 81.6% removal efficiency was attained with an initial SDS concentration of 2.5 mg L^−1^ (Arslan et al., 2018). Notably, studies employed much lower initial SDS concentrations than real-world scenarios. Furthermore, the ECO approach benefits from not requiring the addition nor handling of chemicals.

### 3.3 Understanding the influence of idiosyncrasies of the greywater matrix on surfactant removal

**Fig. 4:**
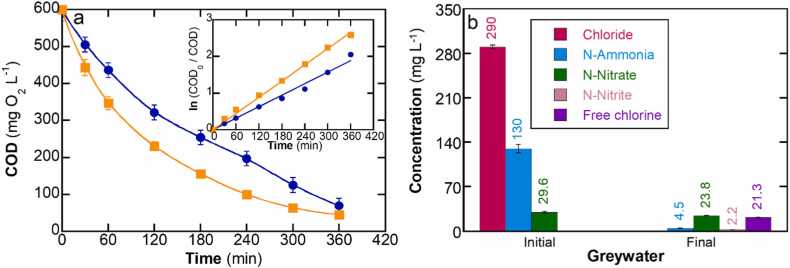
(a) COD removal versus electrolysis time for the treatment of 300 mg L^−1^ of SDS (600 mg L^−1^ COD) in () SDS in water and () model greywater. Inset: kinetic values calculated based on a first-order reaction (b) Evolution of inorganic species present in the model greywater.

The composition of the water matrix exerts a significant influence on ECO. As depicted in Fig. 4a, it is evident that the mineralization exhibited a moderate increase when tested in model greywater. The kinetic constant value (see inset Fig. 4a) was 1.5 times higher when compared to the medium containing only SDS in water (increasing from 4.8 ×10^−3^ min^−1^ to 7.3 ×10^−3^ min^−1^). Greywater contains various inorganic species, as detailed in Table 1, some of which are not electrochemically inert in solution such as chloride, ammonium, and nitrate. These species may generate oxidants in addition to hydroxyl radicals and sulfate radicals, with could explain the enhanced SDS mineralization rate in the model greywater. Among the oxidants that can be generated, active chlorine species, including free chlorine (i.e., Cl_2_, HClO, and ClO^−^) are important contributors. These species are formed through the oxidation of chloride ions, resulting in the formation of chlorine gas (Cl_2_), which readily undergoes hydrolysis to form hypochlorous acid (HClO) and hypochlorite anion (ClO^−^), as depicted in (7), (8), (9) (Ganiyu et al., 2021). Fig. 4b shows that 21.3 mg L^−1^ of free chlorine species (as Cl_2_) were formed from an initial chloride content of 292.6 mg L^−1^. Thus, it is important to note that most of the chloride remains in solution and only part of the initial content is oxidized yielding free chlorine.

(7) 2Cl^−^ → Cl_2_ + 2e^−^

(8) Cl_2_ + H_2_O → HClO + Cl^−^ + H^+^

(9) HClO ⮀ ClO^−^ + H^+^

The initial nitrogen species, namely ammonium and nitrate, exhibited distinct behavior within the electrochemical medium. Notably, there was a significant reduction in ammonia concentration, from 130 mg L^−1^ NH_3_-N to 4.5 mg L^−1^ NH_3_-N. This decrease can be attributed to the reaction of ammonia with free chlorine species, leading to the generation of chloramines that get completely oxidized to nitrogen gas (da Costa et al., 2021; Garcia-Segura et al., 2019). In contrast, nitrate exhibited a more modest change, decreasing from 29.6 mg NO_3_^−^-N to 23.8 mg NO_3_^−^-N. This trend suggests a potential electroreduction process in which nitrate was partially converted to nitrite reaching a concentration > 2.2 mg N-NO_2_^−^. Additionally, nitrate ions within the reaction medium can react with hydroxyl radical, yielding the nitrate radical (Eq. 10). Despite its relatively high redox potential, typically ranging from 2.3 to 2.5 V, the nitrate radical exhibits heightened reaction selectivity, primarily engaging with aromatic compounds (Rayaroth et al., 2022). Consequently, in the context of our study, we can assert that the nitrate radical exerts minimal influence on the mineralization of SDS.

(10) NO_3_^-^ + ^•^OH → NO_3_^•^

### 3.4 Elucidating the degradation pathway of SDS

**Fig. 5:**
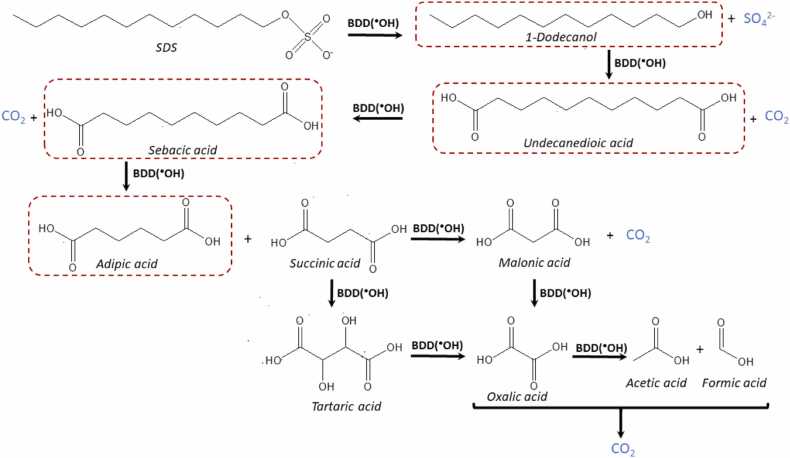
Proposed SDS degradation pathway. Dashed species have been previously identified in trace concentrations as key initial steps on SDS degradation (Lu et al., 2022). Short dicarboxylic species have been detected by ion exclusion chromatography.

Beyond the process of mineralization, it holds paramount importance to comprehensively investigate and identify the byproducts that may arise. In many studies addressing surfactant removal, the formation of byproducts is often overlooked, creating a notable gap in our understanding of the reaction mechanisms. A recent study conducted by Lu et al. (2022) suggested that the first oxidation step involves the cleavage of the sulfonic group yielding 1-dodecanol and sulfate ion. Whereas the subsequent reactions yielding C-11 and C-10 dicarboxylic acids undecanedioic acid and sebacic acid, respectively. Long chain carboxylic acids have been reported to be short-lived during mineralization by electrochemical advanced oxidation process (Barhoumi et al., 2017, Brillas, 2000) and were not detected during our treatment. Further oxidation of sebacic acid induces the scission of C-C bonds yielding shorter length dicarboxylic acids such as adipic acid (C-6) and succinic acid (C-4). The alkyl chain of succinic acid can afterwards be further hydroxylated yielding tartaric acid (C-4) or follow a carbon scission yielding malonic acid (C-3). The final by-products of oxidation induced by electrogenerated hydroxyl radical are the commonly detected species during EAOPs oxalic acid (C-2), acetic acid (C-2) and formic acid (C-1). These three species finally evolve CO_2_ when reaching complete mineralization. The significant detection of these short dicarboxylic acids has not been previously reported during the mineralization of SDS, contributing to complete the elucidation of the degradative pathway of this relevant surfactant by ECO. These species reached values of concentration lower than 2 mg L^−1^ at the end of the ECO treatment. Fig 5.

## 4 Conclusions

This study provided valuable insights into ECO technology for effectively removing SDS in greywater. The selection of anodic materials emerges as a key parameter in the ECO process, with BDD electrodes proving to be the optimal choice, outperforming IrO_2_ and Pt regarding COD abatement rate and energy consumption. The *j* of 100 mA cm^−2^ was identified as the most effective, maximizing the utilization of applied electrical charge while minimizing interference from parasitic reactions. The impact of initial SDS concentration underscores the necessity for a proportional adjustment of reaction time with increasing concentration, revealing that higher concentrations facilitate mass transport, resulting in lower energy consumption. A comprehensive understanding of the influence of the greywater matrix revealed the enhancing effect of inorganic species such as chloride, ammonium, and nitrate on SDS mineralization. The formation of active chlorine species further contributes to improved performance, particularly in model greywater. Finally, the elucidation of the mineralization pathway of SDS identified short-chain carboxylic of different sizes are main reaction intermediates. According to our findings, this study can open doors for new investigations involving greywater treatment and the construction of decentralized equipment that operates with electrochemical technologies. It is important to acknowledge that substantial improvements are essential to realize this objective such as capital cost reduction of electrode materials. Creating a robust electrified prototype that tackles the different challenging components of greywater to enable water reuse is another crucial step for future research and development in decentralized electrochemical water technologies.

## CRediT authorship contribution statement

**Li Qilin:** Writing – review & editing, Visualization, Validation, Project administration, Funding acquisition. **Garcia-Segura Sergio:** Writing – review & editing, Writing – original draft, Visualization, Supervision, Resources, Project administration, Methodology, Funding acquisition, Data curation, Conceptualization. **Lanza Marcos RV:** Writing – review & editing, Visualization, Validation, Funding acquisition. **dos Santos Alexsandro Jhones J.:** Writing – review & editing, Writing – original draft, Visualization, Supervision, Methodology, Investigation, Formal analysis, Data curation. **Shen Hongchen:** Writing – review & editing, Investigation, Formal analysis.

## Declaration of Competing Interest

The authors declare that they have no known competing financial interests or personal relationships that could have appeared to influence the work reported in this paper.

## Data Availability

Data will be made available on request.
